# Linkage analysis between dominant and co-dominant makers in full-sib families of out-breeding species

**DOI:** 10.1590/S1415-47572010000300021

**Published:** 2010-09-01

**Authors:** Alexandre Alonso Alves, Leonardo Lopes Bhering, Cosme Damião Cruz, Acelino Couto Alfenas

**Affiliations:** 1 Departamento de Fitopatologia, Universidade Federal de Viçosa, Viçosa, MGBrazil; 2 Embrapa Agroenergia, Parque Estação Biológica, Brasília, DFBrazil; 3 Departamento de Biologia Geral, Universidade Federal de Viçosa, Viçosa, MGBrazil

**Keywords:** statistical genomics, exogamic populations, recombination frequency and maximum likelihood

## Abstract

As high-throughput genomic tools, such as the DNA microarray platform, have lead to the development of novel genotyping procedures, such as Diversity Arrays Technology (DArT) and Single Nucleotide Polymorphisms (SNPs), it is likely that, in the future, high density linkage maps will be constructed from both dominant and co-dominant markers. Recently, a strictly genetic approach was described for estimating recombination frequency (*r*) between co-dominant markers in full-sib families. The complete set of maximum likelihood estimators for *r* in full-sib families was almost obtained, but unfortunately, one particular configuration involving dominant markers, segregating in a 3:1 ratio and co-dominant markers, was not considered. Here we add nine further estimators to the previously published set, thereby making it possible to cover all combinations of molecular markers with two to four alleles (without epistasis) in a full-sib family. This includes segregation in one or both parents, dominance and all linkage phase configurations.

## Introduction

The first maximum likelihood estimators of recombination frequency for a variety of genetic situations in BC_1_ and F_2_ populations were developed in the early 1950's. For F_2_ with dominant markers, Tan and Fu (2007) recently improved two-point estimates by taking averages from three-point maximum likelihood estimates, whereas Jansen (2009) developed another method for ordering dominant markers by minimizing the number of recombinations between adjacent markers, as a simple alternative to multi-point maximum likelihood. Three-point estimates of recombination frequencies were previously used by Ridout *et al.* (1998) for out-breeding species. Nevertheless, linkage analysis of crosses with out-breeders was first dealt with by Ott (1985); Ritter *et al.* (1990); Arús *et al.* (1994); Ritter and Salamini (1996); Maliepaard *et al.* (1997). Together these papers provided useful formulas for estimating recombination frequency in almost every situation. In some cases, the formulas represent actual estimators, whereas in others they are likelihood equations requiring implementation in numerical maximization methods, such as an EM algorithm, Newton-Raphson, or solved by a graphic method. Recently, in an extensive work with full-sib families, Bhering *et al.* (2008) obtained estimators that differed from those obtained by Maliepaard *et al.* (1997), for recombination frequency of different marker configurations, by using a strictly genetic approach, *i.e.* the expected proportion of each phenotypic class in terms of recombination frequency. Based on the latter, an exogamic population mapping module was implemented in GQMOL (GQMOL, 2009) software, extensively used in Brazil for genetic mapping and QTL analysis. Unfortunately, one particular configuration was not dealt with in the mentioned paper, since distance estimation between dominant markers segregating in a 3:1 ratio and co-dominant markers, was not taken into consideration. With the advent of high-throughput genomic tools, such as the DNA microarray platform, new dominant genotyping technology has been developed, such as DArTs (Wenzl *et al.*, 2004) and SNPs. In the future, it is most likely that high density linkage maps will be constructed from both dominant and co-dominant markers. Such maps will facilitate well-defining the genetic location of functional markers through flanking high-density co-dominant/dominant markers. Nevertheless, due to dominance, the genotype of an individual at a dominant marker is often ambiguous, thereby increasing complexity in analysis. Consequently, the accurate estimation of recombination fractions between dominant markers and between dominant and co-dominant markers, becomes important (Tan and Fu, 2007).

Here, we provide an extension of Bherings work, which enables the estimation of the recombination frequency between dominant markers segregating in a 3:1 ratio, and co-dominant markers in full-sib families. Our estimators and algorithm were developed based on the expected frequencies for each genotypic class. These frequencies were used for building likelihood functions for each possible marker configuration. Based on intrinsic properties and their implementation in free linkage software (GQMOL, 2009), this should be of exceptional use for research groups, whose scope is mapping and the use of molecular markers for selecting monogenic traits, such as disease resistance, plant height, and early flowering, amongst other important dominant traits which are subject to breeding in out-crossing species or constructing high density genetic maps of both dominant and co-dominant markers.

## Methods

###  Estimation of recombination frequency

In full-sib families, markers may vary in the number of segregating alleles (up to four), by one or both parents being heterozygous, markers being dominant or co-dominant, and usually the linkage phases of marker pairs are unknown. Different types of categories and crossings may occur in the general case of multi-allelic systems with four or more alleles (Haseman and Elston, 1972). When considering an A locus with i, j, k and l alleles, there are seven possible types of crosses (Bhering *et al.*, 2008), but only four are considered to be informative, since they segregate for at least one parent. Another particularity of genetic mapping in out-breeding species is that the linkage phase is not known *a priori*, as full-sib families are two generation pedigrees. Thus, one has to considerer four combinations, in order to define the correct linkage phase. Alleles might be linked by coupling to one of the parents and undefined for the other, linked by repulsion to one of the parents and undefined for the other, linked by coupling to both parents, or linked by repulsion to both parents (Maliepaard *et al.*, 1997). Therefore, the correct linkage phase is usually determined *a posteriori* by comparing LOD scores obtained for each combination (Bhering *et al.*, 2008).

When considering these particularities, the estimation of recombination frequency (*r*) in full-sib families may be achieved by using the maximum likelihood method. With this method, the expected frequencies for each genotypic class (*pi*), which are, in turn, dependent on the recombination frequency between markers (*r*), are used to built likelihood functions [L(r;ni)], which, after being maximized for *r*, give the proper estimator for recombination frequency. For this, let the genotypes of two individuals of an outbreed population for a particularly marker, be A_1_A_2_ and A_3_A_4_, respectively. If these two individuals are bred to form a full-sib family the expected segregation pattern is: 1A_1_A_3_:1A_1_A_4_:1A_2_A_3_:1A_2_A_4_. Now, let the genotypes of these same two individuals be B_1_B_2_ and B_3_B_4_ for another marker. If these two individuals are also bred to form a full-sib family the expected segregation pattern is: 1B_1_B_3_:1B_1_B_4_:1B_2_B_3_:1B_2_B_4_.

On considering the haplotypes for the markers in the first parent in the coupling phase, the produced gametes and their frequencies are: f(A_1_B_1_) = f(A_2_B_2_) = (1-r)/2 = P; f(A_1_B_2_) = f(A_2_B_1_) = r/2 = R, whereas for the second parent, the expected gametes and frequencies are: f(A_3_B_3_) = f(A_4_B_4_) = (1-r)/2 = P; f(A_3_B_4_) = f(A_4_B_3_) = r/2 = R.

On now considering gametes produced by these two individuals, 16 genotypic classes are to be expected in the progeny. The genotypic frequencies for these 16 classes are provided in Table S1. If one now considers that B_1_ = B_3_ = B and B_2_ = B_4_ = b, and that BB and Bb are indistinguishable, which typically makes the B marker dominant, the estimation of recombination frequency between these two markers can be made by applying the maximum likelihood method. The likelihood function can be written as:



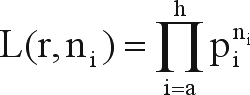


which is

L(r;ni) = [N!/(n_A_!....n_H_!)] x (P^2^+PR+PR)^na^ x (R^2^)^nb^ x (P^2^+PR+R^2^)^nc^ x (PR)^nd^ x (P^2^+PR+R^2^)^ne^ x (PR)^nf^ x (PR+PR+R^2^)^ng^ x (P^2^)^nh^,

and in its simplified form as:

L(r;ni) = λ (1/4-R^2^)^na^(R^2^)^nb^(1/4-PR)^nc^(PR)^nd^ (1/4-PR)^ne^(PR)^nf^(1/4-P^2^)^ng^(P^2^)^nh^

where PP is (1-r)^2^/4, PR is r(1-r)/4, RR is r^2^/4, n_a_ is the total number of individuals with genotypes A_1_A_3_B_, n_b_ is the total number of individuals with genotypes A_1_A_3_bb, n_c_ is the total number of individuals with genotypes A_1_A_4_B_, n_d_ is the total number of individuals with genotypes A_1_A_4_bb, n_e_ is the total number of individuals with genotypes A_2_A_3_B_, n_f_ is the total number of individuals with genotypes A_2_A_3_rr, n_g_ is the total number of individuals with genotypes A_2_A_4_B_, n_h_ is the total number of individuals with genotypes A_2_A_4_bb and N is the total number of individuals.

The estimate of the recombination fraction is then obtained by the usual method of maximizing the logarithm of the likelihood function (Table 1).

However, as previously mentioned, different types of crossings may occur in a full-sib family (Haseman and Elston, 1972). Thus, in order to develop general formulas for estimators of recombination frequency between dominant marker segregating in a 3:1 ratio and co-dominant makers in full-sib families, one has to consider all the different segregation patterns and linkage phases for the co-dominant marker. While the genotypes for the dominant will always be Bb (for both parents), on considering the different types of crosses mentioned above, the genotypes for the co-dominant marker may be: 2 alleles - A_1_A_1_xA_1_A_2_, A_1_A_2_xA_2_A_2_, A_1_A_2_xA_1_A_2_; 3 alleles - A_1_A_1_xA_2_A_3_, A_1_A_2_xA_3_A_3_, A_1_A_2_xA_1_A_3_, A_1_A_2_xA_2_A_3;_ 4 alleles - A_1_A_2_xA_3_A_4_.

So in order to provide an extension of Bherings work which would enable the estimation of recombination frequency between dominant markers segregating in a 3:1 ratio and co-dominant makers in full-sib families we have built likelihood functions to estimate the recombination frequency for each possible marker configuration based on the expected frequencies for each genotypic class as described above (Tables S2 and S3).

###  Average Information content and variance of recombination frequency estimators

Bias and variance are important characteristics describing how close one can get to the true value (Maliepaard *et al.*, 1997). Variances of estimated recombination fractions can be estimated from average information content (Liu, 1997). Within that context, the general formula for estimating information content per observation for any single likelihood parameter (θ) is



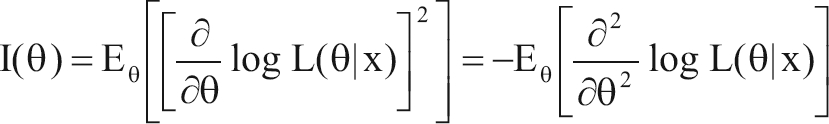


which is -1 times the expectation of the second derivative of the log likelihood function or the support function with respect to the parameter (θ).

The variance of a maximum likelihood estimate from a sample size of N is then:



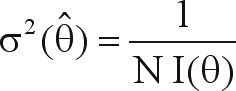


Since the variances of ML-estimators are approximately equal to the inverse of Fisher's information, *i.e.* the expectation of minus the second derivative of the log-likelihood function (Maliepaard *et al.*, 1997 and Schuster and Cruz, 2004), we used this approach to obtain the respective functions.

###  Algorithm integration in GQMOL and mapping procedures

A computer algorithm capable of recognizing the different types of crosses, segregation and linkage phases, and of calculating recombination frequency between dominant markers, as well as the co-dominant markers linked to it based on the likelihood functions here described, was implemented into GQMOL software (GQMOL, 2009). This first requires the construction of an integrated linkage map without the dominant marker, according to traditional methods as described by Ott (1985); Ritter *et al.* (1990); Arús *et al.* (1994); Ritter and Salamini (1996); Maliepaard *et al.* (1997) and Bhering *et al.* (2008). Recombination frequency between the dominant marker and the previously mapped co-dominant marker, according to the likelihood functions here described, is then calculated (see results section). In order to define the correct linkage phase, recombination frequencies are estimated for each of the possible phases predicted in Table S3, and then compared in terms of LOD scores. By comparing scores, the algorithm determines the correct linkage phase, and, in turn, the correct recombination frequency, by identifying the phase and the associated *r* that reached the highest LOD score. After determining the recombination frequency between dominant marker and each of the co-dominant markers, its position on the previously constructed linkage map is defined by traditional alignment methods, such as SARF (Sum of Adjacent Recombination Frequencies) and RCD (Rapid Chain Delineation).

###  Simulation design and testing

Two hundred (200) individuals segregating for 30 loci were generated according to Mendelian inheritance at a given recombination frequency. The simulated genome consisted of 30 markers distributed at an equal distance throughout three linkage groups. Parents were generated randomly, with four alleles in equal frequency - 25%, and markers segregated in various configurations (Haseman and Elston, 1972). To build the simulated map, recombination frequency and LOD scores were calculated using formulas as described by Bhering *et al.* (2008). So as to test the algorithm, data of one specific marker derived from cross A_1_A_2_ x A_1_A_2_ was later re-coded as a dominant marker. Considering that the A1 allele is dominant, data for individuals of genotypes A_1_A_1_ and A_1_A_2_ were retyped as 4, and for individuals A_2_A_2_ were retyped as 2 (4 and 2 are the codes used in GQMOL for the genotypes A_ and aa, respectively). An integrated map without this marker was constructed, as described by Bhering *et al.* (2008). Linkage analysis between the dominant and co-dominant markers was then undertaken, using the functions as presented in Table 1. Comparisons between the *simulated-map* and *algorithm-map* were carried out in terms of marker ordering, distance between markers, total map size, distance variance and stress, in order to evaluate whether the algorithm was efficient as a mapping procedure for dominant markers in full-sib families. A GQMOL simulation module was used for analysis. Simulation was based on 1000 population replicates.

## Results

The genotypic frequencies expected for each marker configuration/linkage phase combination, including those predicted by Haseman and Elston (1972), are given in Table S3. Likelihood functions, as well as estimators of recombination frequency between dominant and co-dominant markers, for all types of crosses and segregations in full-sib families of out-breeding species, are given in Table 1. For practical purposes, it is noteworthy that estimators, which are mainly complex polynomials, have a limited value due to their high degree. However, with GQMOL, it is possible to circumvent this limitation by using a graphic method, so that *r* is calculated directly from likelihood functions. Hence, different values are attributed to *r* (in the 0 to 0.5 interval), and LOD score areas calculated for each value. By plotting these scores on a graph having *r* values in its x-coordinate, and LOD scores in the y-coordinate, the highest LOD score is identified on the graph, and the corresponding *r* value on the abscissa (Schuster and Cruz, 2004).

**Figure 1:**
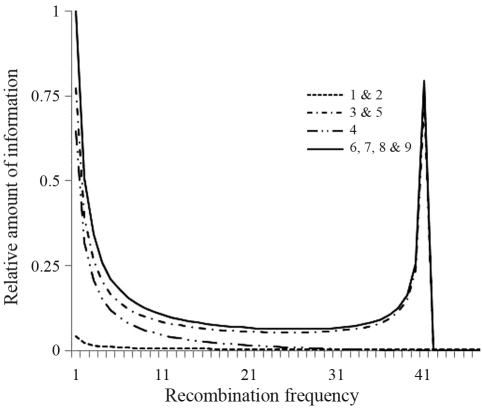
Information content functions relative to all marker configurations involving dominant markers and co-dominant markers in full-sib families of out-breeding species. Configuration *1* refers to crosses A_1_A_1_xA_1_A_2_; A_1_A_1_xA_2_A_3_; A_1_A_2_xA_2_A_2_; A_1_A_2_xA_3_A_3_ in coupling; configuration *2*, to crosses A_1_A_1_xA_1_A_2_; A_1_A_1_xA_2_A_3_; A_1_A_2_xA_2_A_2_; A_1_A_2_xA_3_A_3_ in repulsion; configuration *3* to cross in A1A2xA1A2 coupling, configuration *4* to cross in A_1_A_2_xA_1_A_2_ coupling-repulsion; configuration *5* to cross in A_1_A_2_xA_1_A_2_; configuration *6* to crosses A_1_A_2_xA_1_A_3_; A_1_A_2_xA_2_A_3_ and A_1_A_2_xA_3_A_4_ in coupling; configuration *7* to crosses A_1_A_2_xA_1_A_3_; A_1_A_2_xA_2_A_3_ and A_1_A_2_xA_3_A_4_ in coupling-repulsion; configuration *8* to crosses A_1_A_2_xA_1_A_3_; A_1_A_2_xA_2_A_3_ and A_1_A_2_xA_3_A_4_ in repulsion-coupling and configuration *9* to crosses A_1_A_2_xA_1_A_3_; A_1_A_2_xA_2_A_3_ and A_1_A_2_xA_3_A_4_ in repulsion.

The average information content functions relative to all marker configurations involving dominant markers and co-dominant markers in full-sib families of out-breeding species, *i.e.* different types of crosses, linkage phases, marker configurations and segregations, are presented in Table 2. These functions are useful for evaluating the accuracy of recombination frequency by means of the variance of the estimates. Figure 1 shows that the combinations of dominant and co-dominant markers in configurations *6*, *7*, *8* and *9* provided a relatively large amount of information. These configurations represent crosses between heterozygous individuals which, according to Haseman and Elston (1972), are the most informative (Bhering *et al.* 2008). As to co-dominant markers in configurations *1*, *2*, *3*, *4* and *5* (some of which are equivalent and have the same information content function), the functions provided relatively little information. As in configurations *1* and *2*, half the progeny is absolutely noninformative, the low information content was indeed expected. Nevertheless, although these latter configurations of dominant and co-dominant markers appear to provide little information, the variance of its estimators was quit low. The variances of estimated recombination frequencies (0.05, 0.10 and 0.20), relative to all marker configurations involving dominant markers and co-dominant markers in full-sib families of out-breeding species and different population size, are given in Table 3. Here, one can observe that the highest efficiency is achieved for completely informative co-dominant markers and crosses (configurations *6*, *7*, *8* and *9*), independent of map saturation, and that with adequate population sizes (≥ 200 individuals), even non-completely informative co-dominant markers, together with dominant markers, may be used for constructing maps. However, if expectation is to obtain a less saturated map, ideally only co-dominant markers in configurations *6*, *7*, *8* and *9* should be selected, in order to correctly map dominant markers.

**Figure 2:**
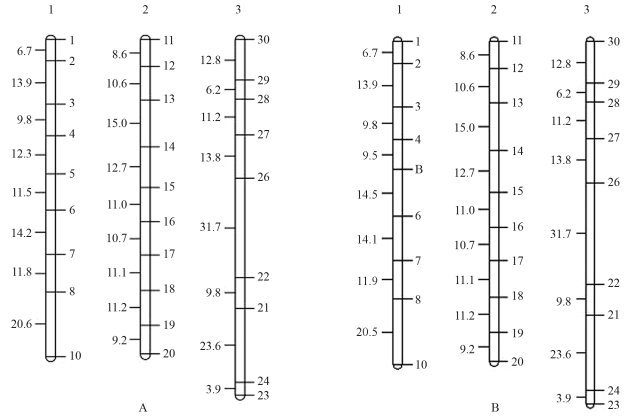
A - simulated genetic map of a full-sib family consisting of three linkage groups and 30 co-dominant markers. B - algorithm-based map of a simulated full-sib family showing the correctly located dominant marker (Marker B - which corresponds to marker 5 in the simulated map).

The algorithm was tested through simulation. The *simulated map* is presented in Figure 2A. Data of one specific locus (marker number 5), derived from cross type A_1_A_2_xA_1_A_2_, and that segregated in a 1:2:1 ratio as evaluated by a chi-square (χ^2^) test, was then re-coded as a dominant marker, as previously described. As expected, linkage analysis without marker 5 data generated a map without the marker itself (data not shown). The linkage map generated with our algorithm and showing marker 5, therein denominated B and correctly located in linkage group 1, is shown in Figure 2B. Comparisons between the *simulated-map* and *algorithm-map* indicated that only linkage group 1 was affected, since linkage groups 2 and 3 remained exactly the same on both maps. This shows that the algorithm did not disturb the alignment of the non-involved linkages groups. Linkage group 1 of the *simulated genome* was 100.82 cM long, whereas the algorithm-based map was 100.98 cM. Marker ordering remained unaltered on the *algorithm map*, with a mean marker distance of 12.63 cM, while on the *simulated map*, the mean distance between markers was 12.60 cM. Map variance increased from 15.97 on the *simulated map* to 17.66 on the *algorithm-based*. Spearman correlation, which measures map ordering consistence, was near 1, thereby indicating that the algorithm, and, in turn, the functions and estimators, were efficient in locating dominant markers. On the other hand, Pearson correlation, which measures correlations between marker distances, was 0.93, thereby also indicating the efficiency of both algorithm and formulas. However, as can be seen in Figures 2A and 2B, the distances between the so called B marker and the 4 and 6 markers are slightly different from those estimated between marker 5 and 4 and 6 on the *simulated map*.

## Discussion

Since most of the computer packages used for genetic mapping are not capable of analyzing out-breeding populations, with the exception of JoinMap (Stam, 1993), over the past years, we have been developing a free genetic software named GQMOL (GQMOL, 2009), apt at analyzing, through genetic mapping, QTL mapping and simulation, not only controlled crosses, but also full-sib and half-sib families. So as to implement an out-breeding population mapping module in GQMOL, Bhering *et al.* (2008) developed likelihood functions and estimators for different marker configurations. However, GQMOL was still inept at estimating the distance between dominant and co-dominant markers. Here, we provide an extension of Bherings work, apt at estimating recombination frequency between a dominant marker segregating in a 3:1 ratio and co-dominant markers in full-sib families. Likelihood functions, used for estimating recombination frequency between the dominant marker and co-dominant markers for each possible marker configuration predicted by Haseman and Elston (1972), were built based on the expected frequencies for each genotype class in a strictly genetic approach. By maximizing the natural logarithm of the log-likelihood functions, the estimators for the recombination frequency between the two markers were obtained. It is noteworthy that our estimators (including those presented in Bhering *et al.* 2008) are quite different from those obtained by Maliepaard *et al.* (1997). These differences are due to the fact that we have applied a strictly genetic approach, rather than a genetic-statistical approach (iterative procedure - EM algorithm) as used by Maliepaard *et al.* (1997). Both methods appear to be equivalent, since the same data packages analyzed by JoinMap and GQMOL resulted in nearly alike integrated maps (AA Alves - unpublished data). However, in situations where the likelihood function is very flat (*i.e.*, the data provide little information due to dominance and markers being in the repulsion phase), the estimates obtained by the EM algorithm may depend on the starting value for recombination frequency. An overall view of likelihood through graphic procedures, or the explicit likelihood function solution, could possibly give rise to recombination frequency associated with the true maximum in a more reliable way. Our method, apart from being simple, may then be more applicable to a wider range of situations than the methods currently available.

A simple simulation approach was chosen to test our algorithm. A simulated full-sib family was generated for the purpose, and data from one specific marker re-coded for dominance, followed by linkage analyses with our algorithm. The dominant marker was correctly located in the linkage map generated with the algorithm, and Spearman and Pearson correlations indicated its efficiency in locating the dominant marker without disturbing nearby markers or other linkage groups. Nevertheless, we noticed that the distances between the dominant marker and those flanking were slightly different from those previously obtained between marker 5 and markers 4 and 6. This was probably due to the loss of information with re-coded data. Whereas three genotypic classes (2 heterozygotes and one homozygote) can be analyzed with co-dominant markers, with dominant markers one can analyze only two (dominant and recessive). This may have affected estimates of recombination frequencies, thereby resulting in different map distances. However, for practical purposes, *e.g.*, MAS - marker assisted selection, bias in distance is not expected to be a problem. Traditional mapping strategies based on co-dominant markers also locate markers near their real position, with an expected bias (Schuster and Cruz, 2004). Our algorithm then, proved to be very fast and precise, and its only prior requirement is a linkage map without the dominant marker constructed following traditional methods as described by Bhering *et al.* (2008) or Maliepaard *et al.* (1997).

As to the accuracy of estimates, it has long been recognized that dominant markers in the repulsion linkage phase supply low linkage information content in F_2_ populations. Nowadays, this problem is receiving additional attention, as high-throughput genomic tools, such as the DNA microarray platform, have lead to the development of up-to-date genotyping procedures resulting in new dominant markers. Novel methods for mapping such markers circumventing this issue have been described (Tan and Fu, 2007; Jansen, 2009). Nevertheless, in full-sib families of out-breeding species, dominant markers appear to be unimpeachable, if used together with co-dominant markers. Our variances estimates for three distinct values of recombination frequency (0.05, 0.10 and 0.20), all marker configurations involving dominant markers and co-dominant markers in full-sib families of out-breeding species and different population size indicates that variances of recombination frequency estimates are very low, ranging from 0.060878318 x 10^-4^ for completely informative markers in a large population (n = 1000) to 8.816327 x 10^-4^ for partially informative markers in a small population (n = 100). These values are very similar to the estimates obtained from co-dominant markers in F_2_ populations, and considerable lower when compared to estimates from both co-dominant and dominant markers in F_2_. For example, for recombination frequencies of 0.05, 0.10 and 0.20, variance estimates for co-dominant markers in an F_2_ of 200 individuals were 1.25 x 10^-4^, 2.53 x 10^-4^ and 5.23 x 10^-4^, respectively (Schuster and Cruz, 2004; Liu, 1997). The variance estimates for co-dominant and dominant markers in the very same F_2_ were 2.47 x 10^-4^, 4.91 x 10^-4^ and 9.69 x 10^-4^, respectively, (Schuster and Cruz, 2004; Liu, 1997). As recombination frequency estimator variance is comprised of two main components, viz., the number of recombination events that created the progeny sample and the (in) ability with which these events can be detected for a certain configuration of two loci, it is reasonable to speculate that the first is defined by recombination frequency itself and progeny size, and the second by the segregation types of loci and linkage phases in the parents (Maliepaard *et al.*, 1997). Hence, although the particularities of out-breeding species (number of segregating alleles and different linkage phases) represent an enormous challenge for genetic mapping, these may, on the other hand, contribute to more accurate estimates of recombination frequency.

Finally, it is noteworthy that Bhering *et al.* (2008) nearly obtained the complete set of maximum likelihood estimators for recombination frequency between molecular markers in full-sib families. With the addition of a further nine, all combinations of molecular markers with two to four alleles (without epistasis) in a full-sib family are now accounted for. This includes segregation in one or both parents, dominance and all linkage phase configurations. In summary, by this paper and Bhering *et al.* (2008), an overview of the whole range of situations of molecular markers in crosses with out-breeding species (full-sib families), has been presented from a genetic perspective. Based on its properties and implementation into free linkage software, our approach should be useful for those interested in using molecular markers for mapping, or as an aid in selecting out-crossing species.

## Supplementary Material

The following online material is available for this article:

Table S1 Genotypic frequencies for a progeny derived from a cross between two fully informative co-dominant markers linked in coupling with four alleles.

Table S2 S2 Probability classes and their respective estimates used in likelihood functions.

Table S3 Genotypic frequencies for progenies derived from crosses between different types of co-dominant markers and a dominant marker for different linkage phases.

This material is made available as part of the online article from http://www.scielo.br.gmb.

## Figures and Tables

**Table 1:** Likelihood functions and expressions for calculating recombination frequency between dominant and co-dominant markers in full-sib families of out-breeding species (different types of crosses, linkage phases - LP and segregations are considered).

Crosses	LP	MC	Likelihood functions	Estimators
A_1_A_1_xA_1_A_2 _A_1_A_1_xA_2_A_3 _A_1_A_2_xA_2_A_2 _A_1_A_2_xA_3_A_3_	C	1	L(r;i) = λ (1/4+P/2)^a^ (R/2)^b^ (1/4+R/2)^c^ (P/2)^d^	r^3^ (N) - r^2^ (2b + 3c + d) - r(a + b - 2(c - d)) + 2b = 0
	R	2	L(r;i) = λ (1/4+R/2)^a^ (P/2)^b^ (1/4+P/2)^c^ (R/2)^d^	r^3^ (N) - r^2^ (3a + b + 2d) + r(2a - 2b - c - d) + 2d = 0
A_1_A_2_xA_1_A_2_	C	3	L(r;i) = λ (1/4-R^2^)^A^ (R^2^)^b^ (1/4+P^2+^R^2^)^c^ (2PR)^d^ (1/4-P^2^)^e^ (P^2^)^f^	2r^7^ (N) - r (2a + 2b + c + d + 4f) - 2r^6^ (4a + 5b + 6c + 5d + 4e + f) + r^5^ (14a + 16b + 10c + 11d + 2(5e + 3f)) - r^4^ (14a + 6b - 8c + 3d + 2(e + 2f)) + r^3^ (4a - 10b - 9c - 2(5d + 4e + f)) + r^2^(14b + 2c + 9d + 4(2e + f)) - 2(2b + d + e) = 0
	C-R	4	L(r;i) = λ (1/4-PR)^a^ (PR)^b^ (1/4+2PR)^c^ (P^2+^R^2^)^d^ (1/4-PR)^e^ (PR)^f^	(2r - 1)(2r 4(N) - 4r 3(N) + r 2(3a + 5b + 4c + 4d + 3e + 5f) - r(a + 3b + 2c + 2d + e + 3f) + b + f) = 0
	R	5	L(r;i) = λ (1/4-P^2^)^a^ (P^2^)^b^ (1/4+P^2+^R^2^)^c^ (2PR)^d^ (1/4-R^2^)^e^ (R^2^)^f^	2r^7^ (N) - r^6^ (4b + c + d + 2(e + f)) - 2r^5^ (4a + b + 6c + 5d + 4e + 5f) + r^4^ (10a + 6b + 10c + 11d + 2(7e + 8f)) - r^3^ (2a + 4b - 8c + 3d + 2(7e + 3f)) - r^2^ (8a + 2b + 9c + 2(5d - 2e + 5f)) + r(8a + 4b + 2c + 9d + 14f) - 2(a + d + 2f) = 0
A_1_A_2_xA_1_A_3_ A_1_A_2_xA_2_A_3_ A_1_A_2_xA_3_A_4_	C	6	L(r;i) = λ (1/4-R^2^)^a^ (R^2^)^b^ (1/4-PR)^c^ (PR)^d^ (1/4-PR)^e^ (PR)^f^ (1/4-P^2^)^g^ (P^2^)^h^	2r^7^ (N) - r^6^ (2a + 2b + c + d + e + f + 4h) - 2r^5^ (4a + 5b + 6c + 5d + 6e + 5f + 4g + h) + r^4^ (14a + 16b + 10c + 11d + 10e + 11f + 2(5g + 3h)) - r^3^ (14a + 6b - 8c + 3d - 8e + 3f + 2(g + 2h)) + r^2^ (4a - 10b - 9c - 10d - 9e - 2(5f + 4g + h)) + r(14b + 2c + 9d + 2e + 9f + 4(2g + h)) - 2(2b + d + f + g) = 0
	C-R	7	L(r;i) = λ (1/4-PR)^a^ (PR)^b^ (1/4-R^2^)^c^ (R^2^)^d^ (1/4- P^2^)^e^ (P^2^)^f^ (1/4-PR)^g^ (PR)^h^	2r^7^ (N) - r^6^ (a + b + 2c + 2d + 4f + g + h) - 2r^5^ (6a + 5b + 4c + 5d + 4e + f + 6g + 5h) + r^4^ (10a + 11b + 14c + 16d + 10e + 6f + 10g + 11h) + r3 (8a - 3b - 14c - 6d - 2e - 4f + 8g - 3h) - r^2^ (9a + 10b - 4c + 10d + 8e + 2f + 9g + 10h) + r(2a + 9b + 14d + 8e + 4f + 2g + 9h) - 2(b + 2d + e + h) = 0
	R-C	8	L(r;i) = λ (1/4-PR)^a^ (PR)^b^ (1/4-P^2^)^c^ (P^2^)^d^ (1/4- R^2^)^e^ (R^2^)^f^ (1/4-PR)^g^ (PR)^h^	2r^7^ (N) - r^6^ (a + b + 4d + 2e + 2f + g + h) - 2r^5^ (6a + 5b + 4c + d + 4e + 5f + 6g + 5h) + r^4^ (10a + 11b + 10c + 6d + 14e + 16f + 10g + 11h) + r^3^ (8a - 3b - 2c - 4d - 14e - 6f + 8g - 3h) - r^2^ (9a + 10b + 8c + 2d - 4e + 10f + 9g + 10h) + r(2a + 9b + 8c + 4d + 14f + 2g + 9h) - 2(b + c + 2f + h) = 0
	R	9	L(r;i) = λ (1/4-P^2^)^a^ (P^2^)^b^ (1/4-PR)^c^ (PR)^d^ (1/4-PR)^e^ (PR)^f^ (1/4-R^2^)^g^ (R^2^)^h^	2r^7^ (N) - r^6^ (4b + c + d + e + f + 2(g + h)) - 2r^5^ (4a + b + 6c + 5d + 6e + 5f + 4g + 5h) + r^4^ (10a + 6b + 10c + 11d + 10e + 11f + 2(7g + 8h)) - r^3^ (2a + 4b - 8c + 3d - 8e + 3f + 2(7g + 3h)) - r^2^ (8a + 2b + 9c + 10d + 9e + 2(5f - 2g + 5h)) + r(8a + 4b + 2c + 9d + 2e + 9f + 14h) - 2(a + d + f + 2h) = 0

**Table 2:** Information content functions relative to all marker configurations involving dominant and co-dominant markers in full-sib families of out-breeding species (different types of crosses, linkage phases - LP, marker configurations -MC and segregations are considered).

Crosses	LP	MC	Function
A_1_A_1_xA_1_A_2_ A_1_A_1_xA_2_A_3_ A_1_A_2_xA_2_A_2_ A_1_A_2_xA_3_A_3_	C	1	- [12r^2^ - 12r - 2] / [r(r + 1)(r - 1)(r - 2)]
	R	2	-[12r^2^ - 12r - 2] / [r(r + 1)(r - 1)(r - 2)]
A_1_A_2_xA_1_A_2_	C	3	-[84r^6^ - 60r^5^ - 250r^4^ + 268r^3^ - 63r^2^ - 70r + 37] / [r(r + 1) (r - 1) (r - 2) (r^2^ - r + 1) (r^2^ + 2r - 1)]
	C-R	4	-[120r^4^ - 240r^3^ + 216r^2^ - 96r + 16] / [r(r - 1) (r^2^ - r + 1) (2r^2^ - 2r + 1)]
	R	5	-[84r^6^ - 60r^5^ - 250r^4^ + 268r^3^ - 63r^2^ - 70r + 37] / [r(r + 1) (r - 1) (r - 2) (r^2^ - r + 1) (r^2^ + 2r - 1)]
A_1_A_2_xA_1_A_3_ A_1_A_2_xA_2_A_3_ A_1_A_2_xA_3_A_4_	C	6	-[4(28r^6^ - 18^^5^ - 90r^4^ + 88r^3^ - 12r^2^ - 27r + 12)] / [r(r + 1) (r - 1) (r - 2) (r^2^ - r + 1 )(r^2^ + 2r - 1)]
	C-R	7	-[112r^6^ - 72r^5^ - 360r^4^ + 352r^3^ - 48r^2^ - 108r + 48] / [r(r + 1) (r - 1) (r - 2) (r^2^ - r + 1) (r^2^ + 2r - 1)]
	R-C	8	-[112r^6^ - 72r^5^ - 360r^4^ + 352r^3^ - 48r^2^ - 108r + 48] / [r(r + 1) (r - 1) (r - 2) (r^2^ - r + 1) (r^2^ + 2r - 1)]
	R	9	-[4(28r^6^ - 18r^5^ - 90r^4^ + 88r^3^ - 12r^2^ - 27r + 12)] / [r(r + 1) (r - 1) (r - 2) (r^2^ - r + 1) (r^2^ + 2r - 1)]

**Table 3:** Variance of estimated recombination frequencies relative to all marker configurations involving dominant and co-dominant markers in full-sib families of out-breeding species and population size.

Marker configuration	Population size (n)				
r = 0.05	100	200	400	800	1000
*1* and 2**	3.78429*	1.892145	0.946072	0.473036	0.378428988
*3* and *5*	0.249117	0.124558	0.062279	0.03114	0.024911692
*4*	0.349641	0.174821	0.08741	0.043705	0.034964109
*6*, *7*, *8* and *9*	0.195527	0.097763	0.048882	0.024441	0.019552669

r = 0.1	100	200	400	800	1000

*1* and *2*	6.107143	3.053571	1.526786	0.763393	0.610714286
*3* and *5*	0.456649	0.228324	0.114162	0.057081	0.045664893
*4*	0.806025	0.403012	0.201506	0.100753	0.080602496
*6*, *7*, *8* and *9*	0.365124	0.182562	0.091281	0.04564	0.036512396

r = 0.2	100	200	400	800	1000

*1* and *2*	8.816327	4.408163	2.204082	1.102041	0.881632653
*3* and *5*	0.731963	0.365981	0.182991	0.091495	0.073196286
*4*	2.462069	1.231034	0.615517	0.307759	0.246206897
*6*, *7*, *8* and *9*	0.608783	0.304392	0.152196	0.076098	0.060878318

*Values were multiplied by 10^4^. **Configuration *1* refers to crosses A_1_A_1_xA_1_A_2_; A_1_A_1_xA_2_A_3_; A_1_A_2_xA_2_A_2_; A_1_A_2_xA_3_A_3_ in coupling; configuration *2*, to crosses A_1_A_1_xA_1_A_2_; A_1_A_1_xA_2_A_3_; A_1_A_2_xA_2_A_2_; A_1_A_2_xA_3_A_3_ in repulsion; configuration *3* to cross in A1A2xA1A2 coupling, configuration *4* to cross in A_1_A_2_xA_1_A_2_ coupling-repulsion; configuration *5* to cross in A_1_A_2_xA_1_A_2_; configuration *6* to crosses A_1_A_2_xA_1_A_3_; A_1_A_2_xA_2_A_3_ and A_1_A_2_xA_3_A_4_ in coupling; configuration *7* to crosses A_1_A_2_xA_1_A_3_; A_1_A_2_xA_2_A_3_ and A_1_A_2_xA_3_A_4_ in coupling-repulsion; configuration *8* to crosses A_1_A_2_xA_1_A_3_; A_1_A_2_xA_2_A_3_ and A_1_A_2_xA_3_A_4_ in repulsion-coupling and configuration *9* to crosses A_1_A_2_xA_1_A_3_; A_1_A_2_xA_2_A_3_ and A_1_A_2_xA_3_A_4_ in repulsion.
