# Supplementary material for: Linkage analysis between dominant and co-dominant makers in full-sib families of out-breeding species
Source: Genet Mol Biol. 2010 Sep 1;33(3):499–506. doi: 10.1590/S1415-47572010000300021 (PMC3036129; doi:10.1590/S1415-47572010000300021)
Supplement: Table S1 — Genotypic frequencies for a progeny derived from a cross between two fully informative co-dominant markers linked in coupling with four alleles. [file gmb-33-3-499-suppl1.pdf]

Table S1 - Genotypic frequencies for a progeny derived from a cross between two fully informative co-dominant markers linked in coupling with four alleles*.

Individuals	Class	Genotypic frequency
A_1A_3 B_1B_3	PP	$(1 - r)^2/4$
A_1A_3 B_1B_4	PR	$r(1 - r)/4$
A_1A_4 B_1B_3	PR	$r(1 - r)/4$
A_1A_4 B_1B_4	PP	$(1 - r)^2/4$
A_1A_3 B_2B_3	PR	$r(1 - r)/4$
A_1A_3 B_2B_4	RR	$r^2/4$
A_1A_4 B_2B_3	RR	$r^2/4$
A_1A_4 B_2B_4	PR	$r(1 - r)/4$
A_2A_3 B_1B_3	PR	$r(1 - r)/4$
A_2A_3 B_1B_4	RR	$r^2/4$
A_2A_4 B_1B_3	RR	$r^2/4$
A_2A_4 B_1B_4	PR	$r(1 - r)/4$
A_2A_3 B_2B_3	PP	$(1 - r)^2/4$
A_2A_3 B_2B_4	PR	$r(1 - r)/4$
A_2A_4 B_2B_3	PR	$r(1 - r)/4$
A_2A_4 B_2B_4	PP	$(1 - r)^2/4$

*$P = (1 - r)/2$; $R = r/2$; $P + R = 0.5$.
