# Supplementary material for: Linkage analysis between dominant and co-dominant makers in full-sib families of out-breeding species
Source: Genet Mol Biol. 2010 Sep 1;33(3):499–506. doi: 10.1590/S1415-47572010000300021 (PMC3036129; doi:10.1590/S1415-47572010000300021)
Supplement: Table S2 — S2 Probability classes and their respective estimates used in likelihood functions. [file gmb-33-3-499-suppl2.pdf]

Table S2 - Probability classes and their respective estimates used in likelihood functions*.

Probabilities	Estimates
P^2	$(1 - r)^2/4$
R^2	$r^2/4$
P/2	(1 - r)/4
R/2	r/4
PR	$(r - r^2)/4$
2PR	$(r - r^2)/2$
$1/4 - P^2$	$(2r - r^2)/4$
$1/4 - R^2$	$(1 - r^2)/4$
1/4 - PR	$(r^2 - r + 1)/4$
1/4 + P/2	(2 - r)/4
1/4 + R/2	(r + 1)/4
1/4 + 2PR	$(2r^2 - 2r - 1)/4$
$PR + PR + P^2$	$(1 - r^2)/4$
$PR + PR + R^2$	$(2r - r^2)/4$
$1/4 + P^2 + R^2$	$(r^2 - r + 1)/4$
$P^2 + R^2$	$(2r^2 - 2r + 1)/4$

*P = (1-r)/2 ; R = r/2; P+R = 0.5.
