# Supplementary material for: Linkage analysis between dominant and co-dominant makers in full-sib families of out-breeding species
Source: Genet Mol Biol. 2010 Sep 1;33(3):499–506. doi: 10.1590/S1415-47572010000300021 (PMC3036129; doi:10.1590/S1415-47572010000300021)
Supplement: Table S3 — Genotypic frequencies for progenies derived from crosses between different types of co-dominant markers and a dominant marker for different linkage phases. [file gmb-33-3-499-suppl3.pdf]

Table S3 - Genotypic frequencies for progenies derived from crosses between different types of co-dominant markers (A locus) and a dominant marker (B locus) for different linkage phases. (In each cross both parents are heterozygous for B locus).

Cross	Segregation	Coupling	Cou-Rep	Rep-Cou	Repulsion
$A_1A_1xA_1A_2$	$A_1A_1B\_$	1/4 + P/2	-	-	1/4 + R/2
	A_1A_1bb	R/2	-	-	P/2
	$A_1A_2B\_$	1/4 + R/2	-	-	1/4 + P/2
	A_1A_2bb	P/2	-	-	R/2
$A_1A_1xA_2A_3$	$A_1A_2B\_$	1/4 + P/2	-	-	1/4 + R/2
	A_1A_2bb	R/2	-	-	P/2
	$A_1A_3B\_$	1/4 + R/2	-	-	1/4 + P/2
	A_1A_3bb	P/2	-	-	R/2
$A_1A_2xA_2A_2$	$A_1A_2B\_$	1/4 + P/2	-	-	1/4 + R/2
	A_1A_2bb	R/2	-	-	P/2
	$A_2A_2B\_$	1/4 + R/2	-	-	1/4 + P/2
	A_2A_2bb	P/2	-	-	R/2
$A_1A_2xA_3A_3$	$A_1A_3B\_$	1/4 + P/2	-	-	1/4 + R/2
	A_1A_3bb	R/2	-	-	P/2
	$A_2A_3B\_$	1/4 + R/2	-	-	1/4 + P/2
	A_2A_3bb	P/2	-	-	R/2
$A_1A_2xA_1A_2$	$A_1A_1B\_$	$1/4 - R^2$	1/4 - PR	-	$1/4 - P^2$
	A_1A_1bb	R^2	PR	-	P^2
	$A_1A_2B\_$	$1/4 + P^2 + R^2$	1/4 + 2PR	-	$1/4 + P^2 + R^2$
	A_1A_2bb	2PR	$P^2 + R^2$	-	2PR
	$A_2A_2B\_$	$1/4 - P^2$	1/4 - PR	-	$1/4 - R^2$
	A_2A_2bb	P^2	PR	-	R^2
$A_1A_2xA_1A_3$	$A_1A_1B\_$	$1/4 - R^2$	1/4 - PR	1/4 - PR	$1/4 - P^2$
	A_1A_1bb	R^2	PR	PR	P^2
	$A_1A_3B\_$	1/4 - PR	$1/4 - R^2$	$1/4 - P^2$	1/4 - PR
	A_1A_3bb	PR	R^2	P^2	PR
	$A_1A_2B\_$	1/4 - PR	$1/4 - P^2$	$1/4 - R^2$	1/4 - PR
	A_1A_2bb	PR	P^2	R^2	PR
	$A_2A_3B\_$	$1/4 - P^2$	1/4 - PR	1/4 - PR	$1/4 - R^2$
	A_2A_3bb	P^2	PR	PR	R^2
$A_1A_2xA_2A_3$	$A_1A_2B\_$	$1/4 - R^2$	1/4 - PR	1/4 - PR	$1/4 - P^2$
	A_1A_2bb	R^2	PR	PR	P^2
	$A_1A_3B\_$	1/4 - PR	$1/4 - R^2$	$1/4 - P^2$	1/4 - PR
	A_1A_3bb	PR	R^2	P^2	PR
	$A_2A_2B\_$	1/4 - PR	$1/4 - P^2$	$1/4 - R^2$	1/4 - PR
	A_2A_2bb	PR	P^2	R^2	PR
	$A_2A_3B\_$	$1/4 - P^2$	1/4 - PR	1/4 - PR	$1/4 - R^2$
	A_2A_3bb	P^2	PR	PR	R^2
$A_1A_2xA_3A_4$	$A_1A_3B\_$	$1/4 - R^2$	1/4 - PR	1/4 - PR	$1/4 - P^2$
	A_1A_3bb	R^2	PR	PR	P^2
	$A_1A_4B\_$	1/4 - PR	$1/4 - R^2$	$1/4 - P^2$	1/4 - PR
	A_1A_4bb	PR	R^2	P^2	PR
	$A_2A_3B\_$	1/4 - PR	$1/4 - P^2$	$1/4 - R^2$	1/4 - PR
	A_2A_3bb	PR	P^2	R^2	PR
	$A_2A_4B\_$	$1/4 - P^2$	1/4 - PR	1/4 - PR	$1/4 - R^2$
	A_2A_4bb	P^2	PR	PR	R^2
